# Effect of High-Pressure GaN Nucleation Layer on the Performance of AlGaN/GaN HEMTs on Si Substrate

**DOI:** 10.3390/ma16093376

**Published:** 2023-04-26

**Authors:** You-Chen Weng, Ming-Yao Hsiao, Chun-Hsiung Lin, Yu-Pin Lan, Edward-Yi Chang

**Affiliations:** 1 College of Photonics, National Yang Ming Chiao Tung University, Tainan 71150, Taiwan; ycweng.c@nycu.edu.tw (Y.-C.W.); teaandtea38@gmail.com (M.-Y.H.); yplan@nycu.edu.tw (Y.-P.L.); 2 Institute of Pioneer Semiconductor Innovation, Industry Academia Innovation School (IAIS), National Yang Ming Chiao Tung University, Hsinchu 30010, Taiwan; chun_lin@nycu.edu.tw; 3 International College of Semiconductor Technology, National Yang Ming Chiao Tung University, Hsinchu 30010, Taiwan

**Keywords:** AlGaN/GaN HEMT, high pressure, nucleation layer

## Abstract

A high-pressure (HP) GaN nucleation layer (NL) was inserted between AlGaN buffer and an unintentionally doped (UID) GaN layer of an AlGaN/GaN HEMT on Si. The XRD and TEM showed that when the V/III ratio was optimized during the HP-GaN NL growth, the edge dislocation density in the HP-GaN NL layer could be reduced significantly. Experimental results exhibited a lower off-state leakage current, higher maximum I_D_ and G_m_ (corresponding to 22.5% and 21.7% improvement, respectively), and lower on-state resistance. These results demonstrate that the electrical properties of the AlGaN/GaN HEMT can be improved through the insertion of a HP-GaN NL.

## 1. Introduction

Due to the wide bandgap nature and high injection velocity at high field of GaN-related material, AlGaN/GaN HEMT structures have been shown to possess a high breakdown field with low specific on-resistance characteristics. They have been widely explored for high power and high RF applications and are considered to be the most promising devices for next generation power and RF electronics [1,2,3,4].

AlGaN/GaN HEMTs can be grown on SiC, sapphire, and Si substrates. Of these, growing GaN on Si has the benefit of fabricating devices on a large wafer (up to 300 mm) [5] with silicon-compatible processes to reduce manufacturing costs. Although growing high quality GaN on Si on large area with good uniformity is very challenging due to large differences in lattice parameters and the thermal expansion coefficient (CTE), many recent reports show that AlGaN/GaN HEMTs structures can be grown on Si with very competitive power performances [6,7,8].

In general, AlGaN/GaN HEMTs on Si for high power applications have been grown using metalorganic chemical vapor deposition (MOCVD) via a thick GaN-based buffer layer (AlGaN, GaN:C, GaN:Fe, and (Al)GaN/AlN superlattice) [9,10,11,12,13,14]. However, the occurrence of cracks during the cooling process certainly limits the overall yield for production. Various nucleation and buffer layers have been employed to grow high quality GaN-based HEMT epitaxial layers on Si without cracks, including a multi-layer AlN structure [15], gradient and step AlGaN buffer [16], (Al)GaN/AlN superlattice (SL) [12], low temperature Al(Ga)N interlayers [11,17,18], and SiN_x_ interlayers [19]. These methods are able to enhance the compressive stress in the top layer to bend the dislocation propagation and thus improve the quality of the film grown on the top. However, there are still many threading dislocations (TD) propagated from Si due to large lattice mismatch. How to bend the dislocations effectively is the most important issue of growing high-quality GaN on Si. T. Egawa [20] reported that a lower TD density could be achieved in the GaN channel by growing GaN buffer in AlGaN/GaN HEMT structure under high chamber pressure. Lower TD density could be associated with less carbon impurity in the film during the growth, resulting in higher current density and better device performance. Despite the lower TD achieved, the off-state leakage current was increased in their study. The mechanism of the existence of a transition from a three-dimensional to two-dimensional growth mode when growing GaN NL at different V/III ratios was first reported by Y. Wong et al. [21]. It was concluded that the improvement in the electrical performance of AlGaN/GaN HEMTs could be achieved through optimized growth conductions.

In this work, a HP-GaN NL grown with optimized V/III ratio was inserted between AlGaN buffer and UID-GaN to improve the overall film quality as well as the electrical characteristics of the devices fabricated. Judging from the data obtained from the in situ reflectance measurement and XRD measurement, the in situ wafer curvature decreased and the GaN film quality improved significantly with this additional HP-GaN NL grown under proper V/III ratio conditions. HR-TEM images showed that the defects were bent due to the insertion of the HP-GaN NL. Measurement results revealed that with the optimized V/III ratio for the growth of HP-GaN NL, the GaN film and the fabricated HEMT showed better electrical characteristics, which was mainly due to the reduction in TD density in the UID-GaN film.

## 2. Materials and Methods

**Figure 1:**
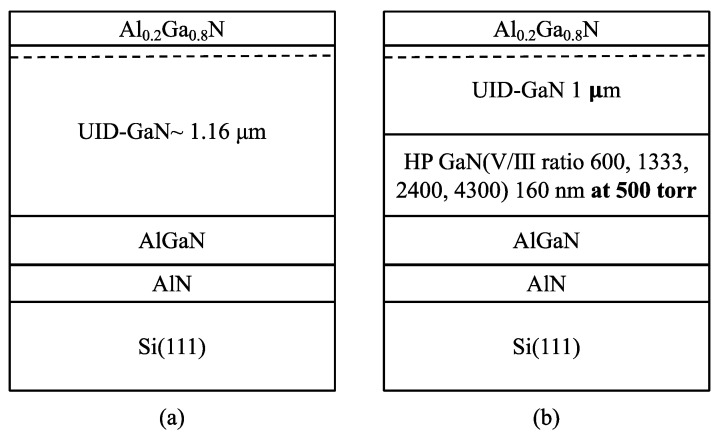
(**a**) Basic schematic diagram of the Al_0.2_Ga_0.8_N/GaN HEMTs structure on Si substrate without the insertion of the HP-GaN NL layer. (**b**) Schematic diagram of the Al_0.2_Ga_0.8_N/GaN HEMTs structure on the Si substrate with the insertion of HP-GaN NL.

Epitaxial structures used in this study were grown on 6-inch Si(111) substrates using the Thomas–Swan metalorganic vapor deposition (MOCVD) system. Trimethylgallium (TMGa), trimethylaluminum (TMAl), and ammonia (NH_3_) were used as the precursors for the Ga, Al, and N elements, respectively. Ultra-high pure H_2_ was used as the carrier gas. The epitaxy structure on the Si substrate grown from bottom to top consisted of the following layers: AlN nucleation layer was first grown, followed by the Al_x_Ga_1−x_N buffer layers, then GaN layer, and finally, the Al_0.2_Ga_0.8_N barrier layer. The GaN layer was grown with and without HP-GaN NL for comparison purposes. The growth conditions of HP-GaN NL were as follows: the reactor pressure was 500 torr, the growth temperature was 1050 °C, and the four samples were deposited at the V/III ratio, varying from 600 to 4300. The NH_3_ flow rate was fixed during the growth of all the samples. All the experiments were monitored by an in situ Laytec EpiCurve^®^ TT system. Figure 1 shows the epitaxial structures without (sample A) and with HP-GaN NL (sample B series). AlN, Al_x_Ga_1−x_N buffer, and the active layers of all samples were grown under the same growth conditions.

The wafer surface temperature, reflectance, and wafer curvature were monitored in situ during the epitaxial growth. The crystal quality of these structures were examined using high-resolution X-ray diffraction (HRXRD, Bede D1) and high-resolution transmission electron microscopy (HR-TEM). A secondary ion mass spectrometer (SIMS) was used to confirm the oxygen and carbon doping levels at each layer of the film. Electron mobility at the 2DEG was measured using Hall measurement (BIO-RAD) and atomic force microscope (AFM) analyses.

After the epitaxial growth, the device fabrication process consisted of four major steps: the ohmic contact formation, the ion implantation isolation, the SiN_x_ passivation, and the gate formation. First, using a contact aligner to define the source and drain regions, the Ti/Al/Ni/Au metals were deposited using an E-gun evaporator, followed by the lift-off process, and ohmic contacts were formed at the source and drain of the device after a rapid thermal annealing (RTA) process at 850 °C for 30 s in N_2_ ambient medium. Then, an ion implantation (^11^B^+^, 190 keV, 3 × 10^13^/cm^3^) isolation process to define the active region of the device was carried out. For passivation, a 20 nm SiN_x_ layer was deposited using plasma enhanced chemical vapor deposition (PECVD). For the gate formation, the gate was fabricated by a contact aligner and the Ni/Au gate metals were deposited by an E-gun evaporator, followed by the lift-off process. The gate length (L_G_), gate-to-drain spacing (L_GD_), and gate-to-source spacing (L_GS_) were 3, 13, and 4 μm, and the gate width (W_G_) was 25 μm, respectively. The electrical characteristics of these devices were measured by Agilent E5270B.

## 3. Results

**Figure 2:**
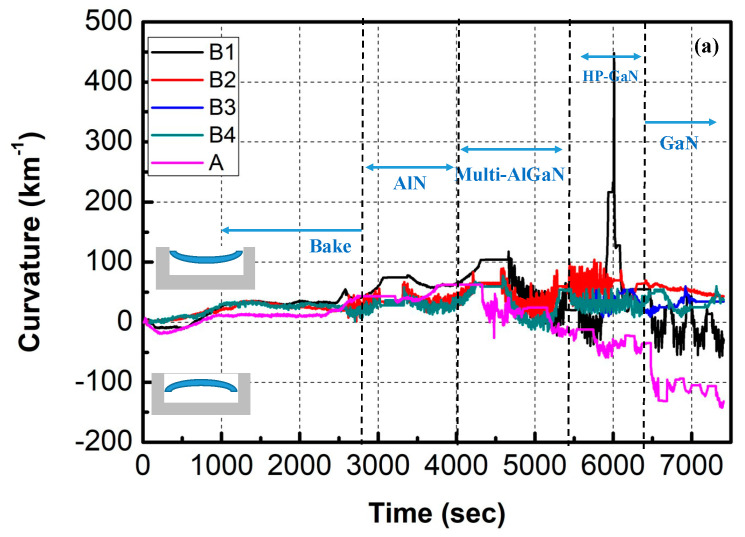

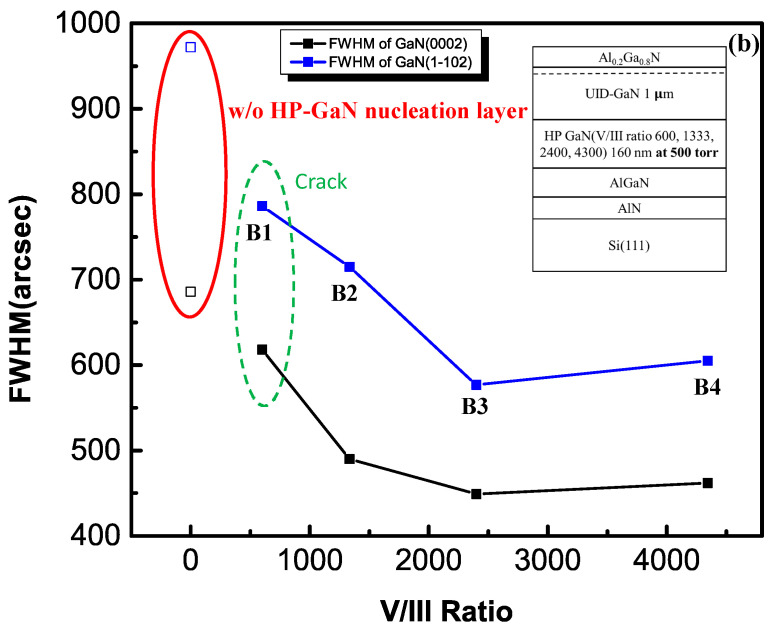
(**a**) In situ measurements of wafer curvature with different V/III ratios of HP-GaN nucleation layer. (**b**) (Color line) XRD omega scan of GaN films grown on various V/III ratios of the HP-GaN nucleation layer.

In this study, wafer curvature was monitored by the Laytec system during the growth of the AlGaN/GaN HEMTs structure. A convex wafer means a negative curvature due to compressive stress, whereas a concave wafer means a positive curvature due to tensile stress. Figure 2a shows the in situ measurement results of wafer curvature under different V/III ratios. It was observed that the tensile stress was introduced continuously during the growing process of the HP-GaN NL, resulting in the change in the bow, which varied from 470 to 75 km^−1^, while the V/III ratio varied from 600 to 4300. It is suggested that the tensile stress during the growth of HP-GaN NL with different V/III ratios can be attributed to the carbon impurity level, growth mode, and growth rate [22].

Figure 2b shows the XRD results of the GaN samples (sample B1, B2, B3, and B4) with HP-GaN NL grown using different V/III ratios and the sample without HP-GaN NL (sample A). The sample with HP-GaN NL showed an improvement of the FWHM of GaN(0002) from 600 to 444 arcsec and the FWHM of GaN(1-102) from 790 to 577 arcsec when the V/III ratio was increased from 600 to 2400 (sample B3).

**Figure 3:**
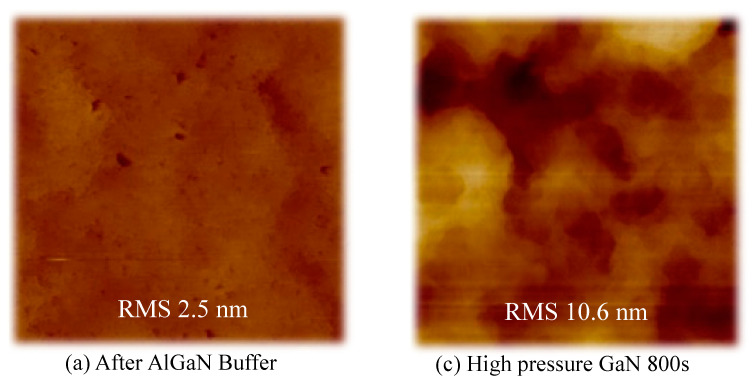

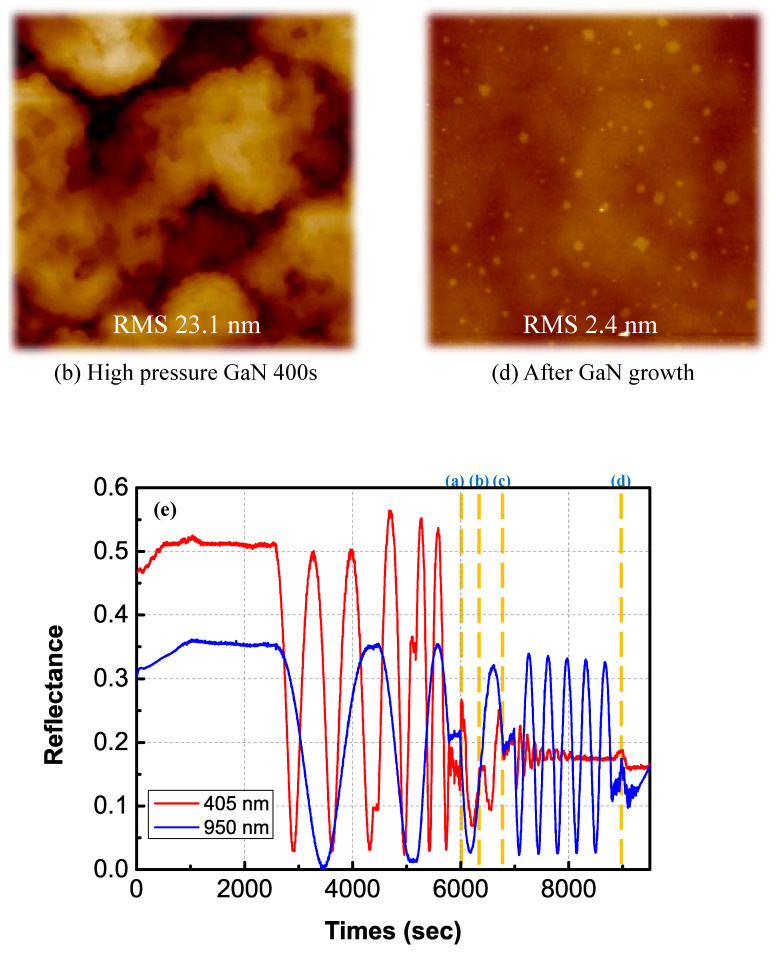
AFM morphology analysis at different growth steps in Sample B3 after (**a**) AlGaN buffer, (**b**) HP-GaN NL 400s, (**c**) HP-GaN NL 800s, and (**d**) the growth of the AlGaN/GaN HEMT structure. (**e**) In situ measurements of reflectance (405 and 950 nm) during the growth of Sample B3.

The morphology of the AlGaN/GaN HEMT epitaxy structure in different growth steps in Sample B3 was measured by AFM analysis, as shown in Figure 3. The measurements in Figure 3a–d were taken after AlGaN buffer, HP-GaN NL 400s, HP-GaN NL 800s, and full AlGaN/GaN HEMT growth, respectively. The 3D to 2D transition occurred during HP-GaN NL growth, Figure 3e showed a 3D to 2D transition time of approximately 800s (observed from 405 nm laser reflectance irregular jitter) of the HP-GaN NL growth. The RMS at 400s of HP-GaN NL was 23.1 nm, showing the 3D growth mode, while the RMS at 800s of HP-GaN NL was 10.6 nm, indicating the transition to 2D growth mode. Finally, AlGaN/GaN HEMT morphology RMS was around 2.4 nm.

**Figure 4:**
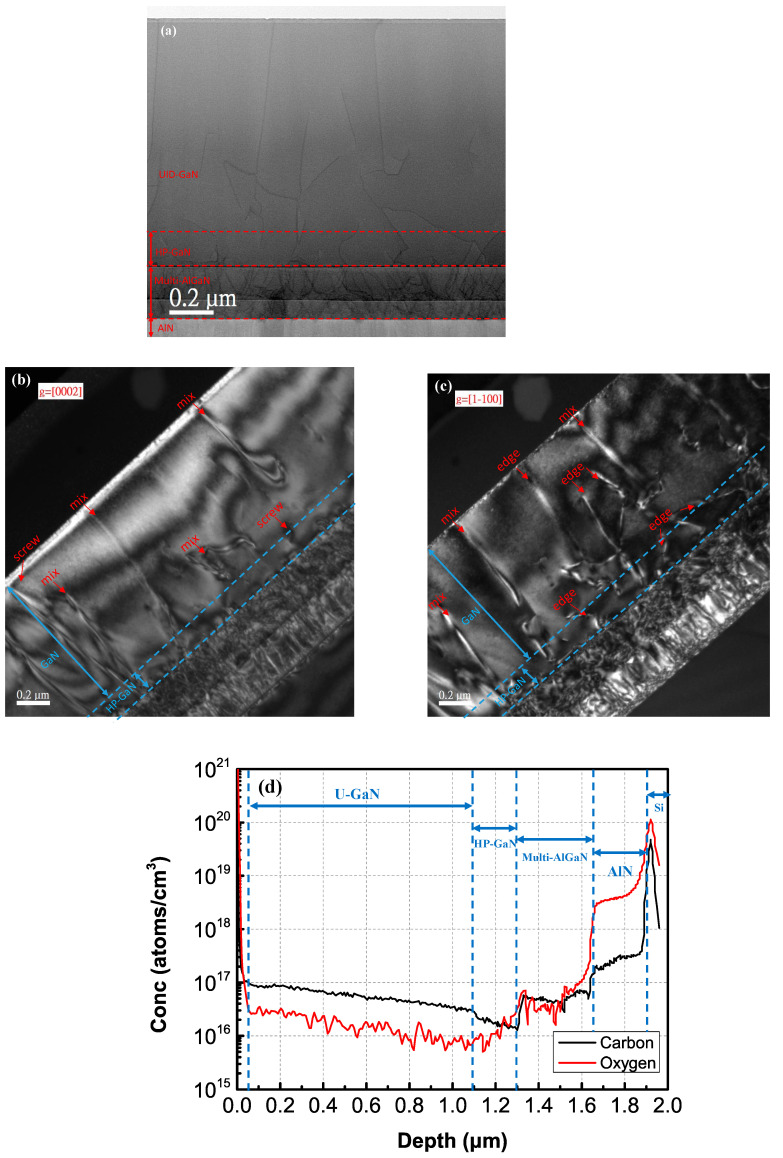
STEM cross-sectional images under bright-field conditions (**a**); cross-sectional TEM images of the whole epitaxial structure under weak-beam dark-field conditions of (**b**) g = [0002] and (**c**) g = [1-100]. (**d**) SIMS depth of O and C impurity from the GaN to AlN buffer in Sample B3. The inset shows HP-GaN between UID-GaN and AlGaN buffer layers.

The HR-TEM images of the complete epitaxial structure of AlGaN/GaN HEMTs with HP-GaN NL (sample B3) inserted between the AlGaN buffer and UID-GaN layer are shown in Figure 4a–c. They showed that the threading dislocations (edge/screw) in the multi-AlGaN buffer layer were effectively bent by HP-GaN NL. Figure 4a shows the weak-beam dark-field TEM image of AlGaN/GaN HEMTs with HP-GaN NL under the g = [0002] condition. The edge dislocations with a Burgers’ vector of b = 1/3<11-20> were invisible, owing to the g*b = 0 criterion. Only screw dislocations with a Burgers’ vector of b = 1⁄4 <0001> and mixed dislocations with a Burgers’ vector of b = 1/3<11-23> were observed. In Figure 4b, the edge and mixed dislocations were shown under the g = [1-100] condition. It again showed that the edge dislocations were reduced by HP-GaN NL immediately after the growth of the multi-AlGaN buffer and revealed the screw dislocation bending in the 3D growth mode GaN grains. In Figure 4c, the HP-GaN NL bent edge dislocations at 3D-2D GaN interface. In both TEM pictures, the HP-GaN NL was seen to effectively bend the threading dislocations, stemming from the multi-AlGaN buffer layers [23,24].

To verify the material compositions of each layer, the SIMS analysis was performed, and the composition profiles of the AlGaN/GaN HEMT device structure with HP-GaN NL are shown in Figure 4d. From the depth profiles, C and O atoms were observed at a distance of around 1 μm below the surface, showing the low O and C content in the UID-GaN and HP-GaN NL. The carbon doping level of HP-GaN NL was around 1.5 × 10^16^ atoms/cc, and similar results were observed in the previous work [25]. The results indicate that lower carbon concentrations in the GaN film could be obtained by increasing the growth pressure.

Hall’s measurement was used to characterize the 2DEG properties of the A and B series samples, as shown in Table 1. At room temperature, the mobility for the A and B series samples were higher than 1500 cm^2^V^−1^s^−1^, and the sheet carrier concentrations were higher than 8 × 10^12^ cm^−2^. Among them, Sample B3 showed the lowest sheet resistance. The low sheet resistance of Sample B3 can be attributed to the lower defect density and impurity concentration. According to Weimann et al. [26], edge dislocation controls the electron mobility in the two-dimensional electron gas channel. Lower mobility observed for Sample A at 77k can be explained by a higher density of edge dislocations. The dangling bonds along edge dislocation play a role as electron acceptors. Therefore, the channel is filled with trapped electrons and the free carrier mobility is reduced in 2DEG. Furthermore, Sample B3 demonstrated a significantly higher mobility of 7770 cm^2^V^−1^s^−1^ with low Rs of 93.54 Ω/sq at 77k. At low temperatures, the effect of crystal defects on mobility became pronounced as there was less phonon scattering. The high electron mobility of Sample B3 could be due to the high-quality GaN film with low dislocation density, as well as lower impurity concentrations [27].

**Figure 5:**
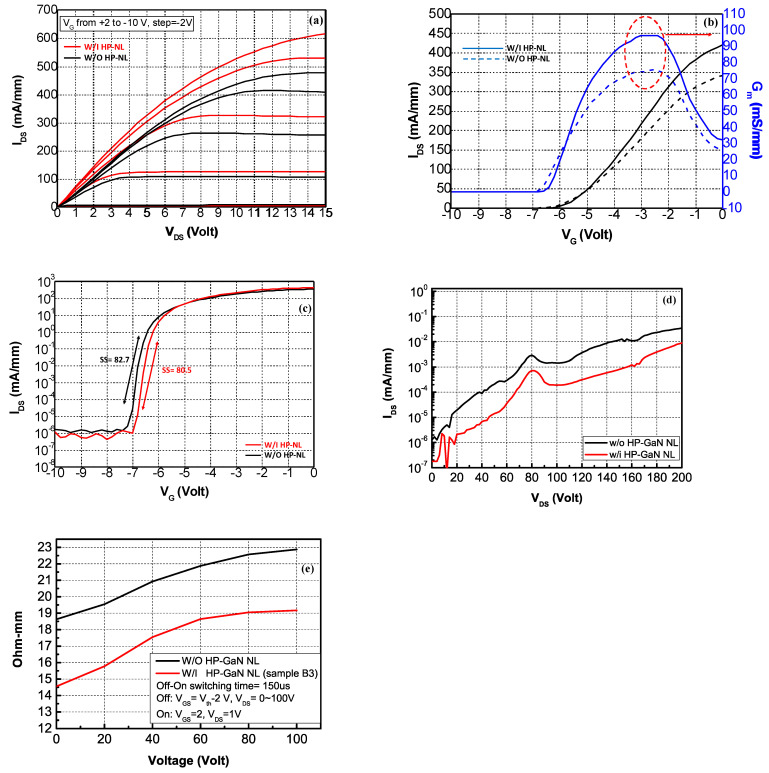
(**a**) I_D_−V_D_ of Sample A and Sample B3, V_g_ from +2 to −10 V, step = −2 V (V_DS_ from 0 to 15 V, step = 0.1 V); (**b**) I_D_−G_m_ electron characteristics of Sample A(dashed lines) and Sample B3(solid lines) (V_G_ from −10 to 0 V, step = 0.1 V, V_DS_ keep at 10 V); (**c**) subthreshold characteristics of Sample A and Sample B3; (**d**) device characteristic of off-state leakage current, V_D_ from 0 to 200 V, step = 0.5 V. V_G_ = −8 V; (**e**) dynamic R_on_ at different drain bias voltages, V_DS_ from 0 to 100 V, step = 20 V.

Figure 5 shows the DC characteristics of the HEMT devices (Sample A and Sample B3) with gate-to-drain spacing (L_GD_) of 13 μm. The trend of the magnitude of the drain currents of the devices is in agreement with the sheet resistance (R_s_) values of the devices. Devices for all samples can be completely pinched-off. The current density of Sample A and Sample B3 were 450 and 545 mA/mm, respectively, when biased at V_G_ = 2 V and V_D_ = 10 V (as shown in Figure 5a). The transfer characteristics of Sample A and Sample B3 with V_D_ = 10 V are plotted in Figure 5b, The threshold voltage (V_th_) of sample B3 (V_th_ = −6.5 V) is less negative than Sample A (V_th_ = −6.9 V). The maximum G_m_ of Sample A and Sample B3 were 75.6 and 96.6 mS/mm, respectively. When the drain currents were compared on the base of the gate over-drive voltage (V_G_-V_th_), the drain current density of Sample B3 was 404 mA/mm when the gate over-drive (V_G_-V_th_) was 6V, which is higher than the current density of the Sample A (I_DS_ = 313 mA/mm). Higher current density (22%) at the same gate over-drive voltage, for Sample B3, indicates lower TDs and lower impurities in the sample, resulting in higher electron mobility. In addition, the subthreshold characteristics of Sample A and Sample B3 are shown in Figure 5c, and the subthreshold slopes (SSs) were 82.7 and 80.5 mV/dec., respectively, and the device off-state leakage current at V_G_ = −10 V for the Sample A and Sample B3 were plotted in Figure 5d. The device off-state breakdown voltage for Sample A and Sample B3 were 72 V and 158 V (as defined by the leakage current of 1 μA/mm). There was a 5 to 10 times significant reduction in the off-state leakage current for Sample B3 as compared with Sample A, indicating lower TDs at the buffer layer. The device dynamic R_on_ characteristics of Sample A and B3 were shown in Figure 5e (from 0 to 100 V, with step of 20 V). Sample B3 had lower R_on_ due to lower defect density, lower traps, and more carrier density in the 2DEG channel.

It is obvious that the device with HP-GaN NL exhibits higher device performances, lower off-state leakage current, and better R_on_ stability under V_D_ stress. This is mainly due to the suppression of the leakage path from the AlGaN buffer by HP-GaN NL. In summary, compared to other groups, HP-GaN NL inserts between U-GaN and AlGaN buffer were able to improve device performance and maintain off-state breakdown voltage [20]. It was thus concluded that the mitigation of the threading dislocation density from the buffer layer was essential for the device to be used for electronic applications.

## 4. Conclusions

In this work, the AlGaN/GaN HEMT with a high-pressure GaN nucleation layer on Si was investigated for electronic applications. Compared to regular HEMT without HP-GaN NL, HEMT with HP-GaN NL has better film quality, higher mobility, lower off-state leakage current, higher current density (22.5% increase in same over-drive voltage), higher G_m_ (21.7% increase), and lower R_on_ (15% decrease). These are due to the HP-GaN NL, effectively bending the threading dislocations and achieving the improvement of the film crystallinity through the insertion of HP-GaN NL. These results show that the AlGaN/GaN HEMT with HP-GaN NL is suitable for electronic applications.

## Figures and Tables

**Table 1:** Electrical properties (sheet resistance, 2DEG N_s_ concentration, 2DEG mobility) of A and B-series samples.

Electrical Property						
	Rs (Ω/sq)		Ns (10^13^ cm^2^/V·s)		Mobility (cm^2^/V·s)	
**Temperature**	**RT**	**77k**	**RT**	**77k**	**RT**	**77k**
**A**	503.4	167.7	−0.82	−0.854	1550	4370
**B2**	491	131.2	−0.832	−0.827	1531	5970
**B3**	463.5	93.5	−0.8495	−0.859	1586	7770
**B4**	465.8	105.5	−0.874	−0.854	1536	6920

## Data Availability

The data presented in this paper are available on request from the corresponding author.

## References

[1] Mishra U.K., Likun S., Kazior T.E., Wu Y.-F. (2008). GaN-Based RF Power Devices and Amplifiers. Proc. IEEE.

[2] Chen K.J., Haberlen O., Lidow A., Tsai C.L., Ueda T., Uemoto Y., Wu Y. (2017). GaN-on-Si Power Technology: Devices and Applications. IEEE Trans. Electron Devices.

[3] Ishida M., Ueda T., Tanaka T., Ueda D. (2013). GaN on Si Technologies for Power Switching Devices. IEEE Trans. Electron Devices.

[4] Geens K., Li X., Zhao M., Guo W., Wellekens D., Posthuma N., Fahle D., Aktas O., Odnoblyudov V., Decoutere S. 650 V p-GaN Gate Power HEMTs on 200 mm Engineered Substrates. Proceedings of the2019 IEEE 7th Workshop on Wide Bandgap Power Devices and Applications (WiPDA).

[5] Then H.W., Radosavljevic M., Koirala P., Beumer M., Bader S., Zubair A., Hoff T., Jordan R., Michaelos T., Peck J. Scaled Submicron Field-Plated Enhancement Mode High-K Gallium Nitride Transistors on 300mm Si(111) Wafer with Power FoM (R_ON_ xQ_GG_) of 3.1 mohm-nC at 40V and f_T_/f_MAX_ of 130/680GHz. Proceedings of the 2022 International Electron Devices Meeting (IEDM).

[6] Amano H., Baines Y., Beam E., Borga M., Bouchet T., Chalker P.R., Charles M., Chen K.J., Chowdhury N., Chu R. (2018). The 2018 GaN power electronics roadmap. J. Phys. D Appl. Phys..

[7] Egawa T. Heteroepitaxial Growth and Power Devices Using AlGaN/GaN HEMT on 200 mm Si (111) Substrate. Proceedings of the 2013 IEEE Compound Semiconductor Integrated Circuit Symposium (CSICS).

[8] Rowena I.B., Selvaraj S.L., Egawa T. (2011). Buffer Thickness Contribution to Suppress Vertical Leakage Current with High Breakdown Field (2.3 MV/cm) for GaN on Si. IEEE Electron Device Lett..

[9] Choi Y.C., Pophristic M., Cha H.-Y., Peres B., Spencer M.G., Eastman L.F. (2006). The Effect of an Fe-doped GaN Buffer on off-State Breakdown Characteristics in AlGaN/GaN HEMTs on Si Substrate. IEEE Trans. Electron Devices.

[10] Chevtchenko S.A., Cho E., Brunner F., Bahat-Treidel E., Würfl J. (2012). Off-state breakdown and dispersion optimization in AlGaN/GaN heterojunction field-effect transistors utilizing carbon doped buffer. Appl. Phys. Lett..

[11] Hsiao Y.-L., Chang C.-A., Chang E.Y., Maa J.-S., Wang Y.-J., Weng Y.-C., Chang C.-T. (2014). Material growth and device characterization of AlGaN/GaN single-heterostructure and AlGaN/GaN/AlGaN double-heterostructure field effect transistors on Si substrates. Appl. Phys. Express.

[12] Selvaraj S.L., Suzue T., Egawa T. (2009). Breakdown Enhancement of AlGaN/GaN HEMTs on 4-in Silicon by Improving the GaN Quality on Thick Buffer Layers. IEEE Electron Device Lett..

[13] He X., Feng Y., Yang X., Wu S., Cai Z., Wei J., Shen J., Huang H., Liu D., Chen Z. (2022). Step-Graded AlGaN vs superlattice: Role of strain relief layer in dynamic on-resistance degradation. Appl. Phys. Express.

[14] Tajalli A., Meneghini M., Besendörfer S., Kabouche R., Abid I., Püsche R., Derluyn J., DeGroote S., Germain M., Meissner E. (2020). High Breakdown Voltage and Low Buffer Trapping in Superlattice GaN-on-Silicon Heterostructures for High Voltage Applications. Materials.

[15] Lin K.-L., Chang E.-Y., Hsiao Y.-L., Huang W.-C., Li T., Tweet D., Maa J.-S., Hsu S.-T., Lee C.-T. (2007). Growth of GaN film on 150mm Si (111) using multilayer AlN∕AlGaN buffer by metal-organic vapor phase epitaxy method. Appl. Phys. Lett..

[16] Lin K.-L., Chang E.-Y., Hsiao Y.-L., Huang W.-C., Luong T.-T., Wong Y.-Y., Li T., Tweet D., Chiang C.-H. (2010). Effects of Al_x_Ga_1−x_N interlayer for GaN epilayer grown on Si substrate by metal-organic chemical-vapor deposition. J. Vac. Sci. Technol. B.

[17] Fritze S., Drechsel P., Stauss P., Rode P., Markurt T., Schulz T., Albrecht M., Bläsing J., Dadgar A., Krost A. (2012). Role of low-temperature AlGaN interlayers in thick GaN on silicon by metalorganic vapor phase epitaxy. J. Appl. Phys..

[18] Dadgar A., Hempel T., Bläsing J., Schulz O., Fritze S., Christen J., Krost A. (2011). Improving GaN-on-silicon properties for GaN device epitaxy. Phys. Status Solidi C.

[19] Hertkorn J., Brückner P., Thapa S., Wunderer T., Scholz F., Feneberg M., Thonke K., Sauer R., Beer M., Zweck J. (2007). Optimization of nucleation and buffer layer growth for improved GaN quality. J. Cryst. Growth.

[20] Selvaraj J., Selvaraj S.L., Egawa T. (2009). Effect of GaN Buffer Layer Growth Pressure on the Device Characteristics of AlGaN/GaN High-Electron-Mobility Transistors on Si. Jpn. J. Appl. Phys..

[21] Wong Y.-Y., Chang E.Y., Huang W.-C., Lin Y.-C., Tu Y.-Y., Chen K.-W., Yu H.-W. (2014). Effects of initial GaN growth mode on the material and electrical properties of AlGaN/GaN high-electron-mobility transistors. Appl. Phys. Express.

[22] Fu Y., Gulino D.A., Higgins R. (2000). Residual stress in GaN epilayers grown on silicon substrates. J. Vac. Sci. Technol. A.

[23] Koleske D.D., Wickenden A.E., Henry R.L., Twigg M.E., Culbertson J.C., Gorman R.J. (1998). Enhanced GaN decomposition in H2 near atmospheric pressures. Appl. Phys. Lett..

[24] Twigg M.E., Henry R.L., Wickenden A.E., Koleske D.D., Culbertson J.C. (1999). Nucleation layer microstructure, grain size, and electrical properties in GaN grown on a-plane sapphire. Appl. Phys. Lett..

[25] Wickenden A., Koleske D., Henry R., Twigg M., Fatemi M. (2003). Resistivity control in unintentionally doped GaN films grown by MOCVD. J. Cryst. Growth.

[26] Weimann N.G., Eastman L.F., Doppalapudi D., Ng H.M., Moustakas T.D. (1998). Scattering of electrons at threading dislocations in GaN. J. Appl. Phys..

[27] Wong Y.-Y., Chang E.Y., Yang T.-H., Chang J.-R., Ku J.-T., Hudait M.K., Chou W.-C., Chen M., Lin K.-L. (2010). The Roles of Threading Dislocations on Electrical Properties of AlGaN/GaN Heterostructure Grown by MBE. J. Electrochem. Soc..

